# Hepatic Brucellosis Mimicking Malignancy in a Cirrhotic Patient: A Rare Case With Atypical Imaging Characteristics

**DOI:** 10.7759/cureus.91940

**Published:** 2025-09-09

**Authors:** Abdulrahman A Almalaq, Hisham M Kardaman, Fuad H Mohammad, Majed S Alzahrani, Nasser M AL Masri

**Affiliations:** 1 Gastroenterology and Hepatology, Prince Sultan Military Medical City, Riyadh, SAU; 2 Hepatology, Prince Sultan Military Medical City, Riyadh, SAU; 3 Internal Medicine, Prince Sultan Military Medical City, Riyadh, SAU

**Keywords:** brucella melitensis, brucellosis, case report, cirrhosis, differential diagnosis, hepatic brucelloma, imaging, liver lesions, microabscess, zoonosis

## Abstract

Brucellosis is a zoonotic infection that can present with a wide range of systemic manifestations. Hepatic involvement is usually mild; however, in rare cases, it can result in focal lesions such as brucellar microabscesses or brucellomas. These may closely mimic hepatic malignancies or other infectious etiologies on imaging, particularly in patients with underlying chronic liver disease, posing a significant diagnostic challenge. We report the case of a 55-year-old Saudi male with a history of type 2 diabetes mellitus, hypertension, and cirrhosis secondary to metabolic dysfunction-associated steatotic liver disease (MASLD), who presented with hematemesis and intermittent fever. Laboratory findings revealed elevated aminotransferases, raised inflammatory markers, and positive serology for *Brucella melitensis*. Imaging demonstrated multiple small ring-enhancing hepatic lesions, initially raising concern for malignancy or disseminated infection. PET-CT showed no hypermetabolic activity, making malignancy less likely. A liver biopsy confirmed advanced fibrosis consistent with cirrhosis, without evidence of malignancy or organized abscess formation. Given the clinical and radiologic context, a multidisciplinary team initiated antimicrobial therapy with gentamicin for two weeks, in combination with doxycycline and trimethoprim-sulfamethoxazole for three months. Follow-up imaging showed significant resolution of the hepatic lesions. This case highlights the diagnostic complexity of hepatic brucellosis in cirrhotic patients, where radiological findings may mimic hepatocellular carcinoma or metastases. Despite advancements in imaging, definitive diagnosis often requires histologic confirmation and clinical correlation. The case underscores the importance of considering brucellosis in the differential diagnosis of hepatic lesions, especially in endemic regions or in patients with exposure risk factors. Early diagnosis and appropriate antimicrobial therapy can lead to complete resolution and avoid unnecessary interventions for presumed malignancy.

## Introduction

Brucellosis is a globally distributed zoonotic infection caused by the *Brucella* genus, typically transmitted through unpasteurized dairy products or direct animal contact. While it most commonly manifests with fever, arthralgia, and hepatosplenomegaly, hepatic involvement is often mild, with nonspecific biochemical or radiologic findings. However, in rare instances, *Brucella* can cause focal hepatic complications, such as hepatic abscesses or brucellomas [1].

Hepatic brucelloma, first described decades ago, remains a rare but significant complication of chronic brucellosis. It is characterized by granulomatous inflammation and necrosis, often forming pseudo-tumoral lesions or microabscesses [2]. Such lesions are typically discovered during evaluation for prolonged fever, hepatomegaly, or abnormal liver function tests in endemic regions [3]. Radiologically, hepatic brucellomas may appear similar to malignancies or tuberculous granulomas, frequently necessitating biopsy for definitive diagnosis [4-6]. Conventional imaging modalities such as ultrasound, CT, and MRI are essential for detecting lesions but often lack specificity [7,8].

Several reports have highlighted diagnostic delays and therapeutic challenges associated with hepatic brucellomas and abscesses. These lesions may remain culture-negative, requiring diagnosis via serologic tests and histopathology [9]. In some cases, hepatic involvement was the initial or sole manifestation of brucellosis, underscoring the need for heightened clinical suspicion in endemic areas [5]. Furthermore, hepatic brucellosis may present atypically with features such as cholestasis, liver failure, or sepsis, mimicking a wide range of hepatic diseases [4,6,10].

Timely diagnosis and appropriate antibiotic therapy can lead to resolution in most cases, although surgical or percutaneous drainage may be required in large or refractory abscesses [8]. This case report contributes to the growing literature by presenting a diagnostically challenging case of hepatic brucellosis mimicking malignancy, highlighting the importance of considering *Brucella* in the differential diagnosis of hepatic lesions in endemic regions.

## Case presentation

A 55-year-old non-smoking Saudi male with an established diagnosis of type 2 diabetes mellitus, hypertension, and liver cirrhosis secondary to metabolic dysfunction-associated steatotic liver disease (MASLD) presented to the emergency department with a history of intermittent fever and hematemesis. The fever had been ongoing for several weeks, characterized by nocturnal spikes associated with chills, night sweats, and generalized fatigue. One day prior to the presentation, he developed an episode of hematemesis consisting of several cups of fresh blood, which prompted his hospital visit. He reported frequent consumption of unpasteurized milk and regular contact with livestock, particularly sheep.

On examination, his body temperature was 38.3°C, while other systemic findings were unremarkable. There were no clinical signs of ascites, hepatic encephalopathy, or peripheral stigmata of advanced chronic liver disease. Initial laboratory evaluation revealed leukocytosis, microcytic hypochromic anemia, and mild thrombocytopenia. Liver function tests revealed marked elevations in aminotransferases, accompanied by a hepatotoxic pattern rather than a cholestatic pattern. Inflammatory markers were significantly raised, with elevated erythrocyte sedimentation rate and C-reactive protein. Tumor markers were unremarkable, except for an elevated CA 19-9 level. Blood and extended blood cultures showed no growth. Viral hepatitis and autoimmune workup, including hepatitis A IgM, hepatitis B surface antigen, hepatitis C antibody, antimitochondrial antibody, and smooth muscle antibody, was negative. Prothrombin time (PT) and international normalized ratio (INR) were within normal limits (Table 1).

**Table 1:** Initial Laboratory Results Abbreviations: WBC, white blood cell count; Hgb, hemoglobin; HCT, hematocrit; MCV, mean corpuscular volume; PLT, platelet count; AST, aspartate aminotransferase; ALT, alanine aminotransferase; ALP, alkaline phosphatase; GGT, gamma-glutamyl transferase; AFP, alpha-fetoprotein; BUN, blood urea nitrogen; ESR, erythrocyte sedimentation rate; CRP, C-reactive protein.

Laboratory Test	Value	Reference Range
White Blood Cell Count	14.89	4.0–11.0 × 10^9^/L
Hemoglobin	88	125–180 g/L
HCT	0.276	40–54%
MCV	75.4	80–100 fL
Platelet Count	120	150–450 × 10^9^/L
AST	962	5–40 U/L
ALT	1134	0–41 U/L
ALP	134	40–129 U/L
GGT	187	8–61 U/L
Total Bilirubin	9	3–20 µmol/L
Albumin	40	35–50 g/L
AFP	2.7	<10 ng/mL
Creatinine	74	62–106 µmol/L
BUN	6.6	2.5–7.1 mmol/L
ESR	66	0–15 mm/hr
CRP	57.1	0.0–6.0 mg/L

Esophagogastroduodenoscopy (EGD) was performed, which revealed three large grade III esophageal varices, one of which showed stigmata of recent hemorrhage with a red wale sign. Endoscopic variceal band ligation was successfully performed. Additionally, mild portal hypertensive gastropathy was noted, with a characteristic mosaic-like mucosal pattern, areas of erythema, and contact friability. No gastric varices were identified.

Given the background of cirrhosis and markedly elevated liver enzymes, a contrast-enhanced CT scan of the abdomen was performed to further evaluate the hepatic parenchyma and exclude complications such as hepatocellular carcinoma, vascular thrombosis, or infectious collections.

**Figure 1:**
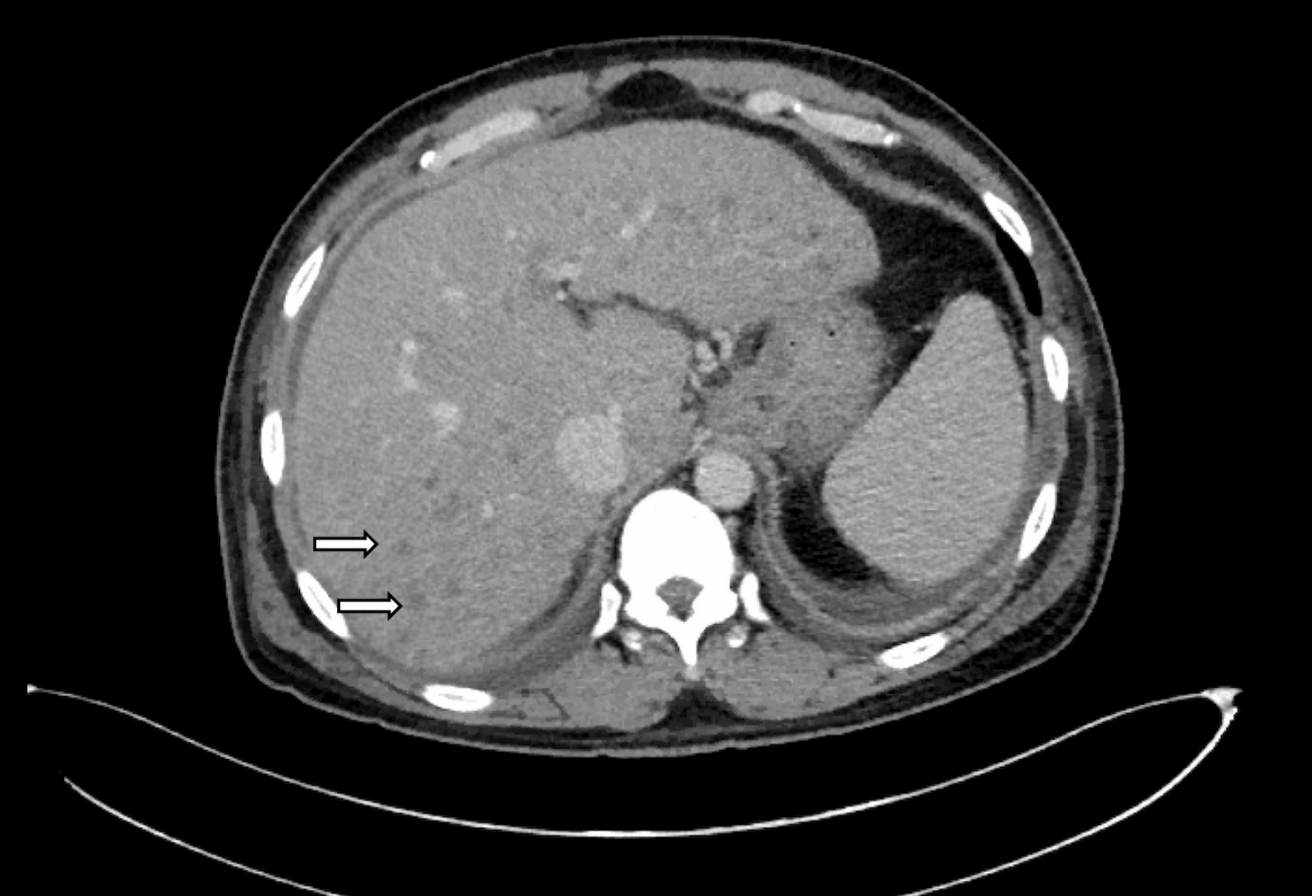
Contrast-enhanced CT abdomen (portal venous phase) shows multiple ring-enhancing lesions throughout the liver (arrows). No central necrosis or washout was evident.

A contrast-enhanced CT abdomen (Figure 1) demonstrated a cirrhotic liver with numerous ring-enhancing lesions involving all hepatic segments. No central washout was observed. Given the morphology of the lesions, further characterization was pursued with liver MRI.

**Figure 2:**
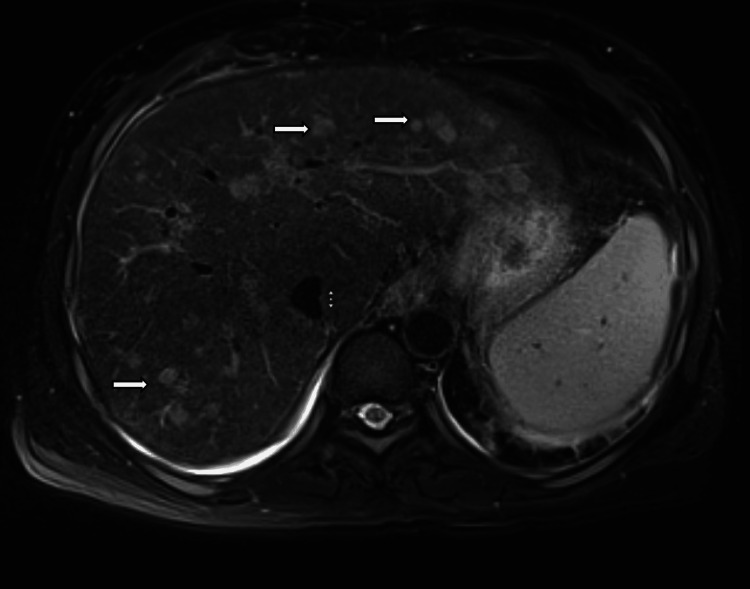
MRI liver with hepatobiliary protocol. Post-contrast axial images show multiple bilobed hepatic lesions with peripheral ring enhancement (arrows), without arterial hyperenhancement or delayed washout.

Liver MRI using a dedicated hepatobiliary protocol (Figure 2) confirmed the presence of bilobed ring-enhancing lesions, which did not demonstrate arterial phase hyperenhancement or delayed washout. Notably, some lesions exhibited restricted diffusion in diffusion-weighted imaging (DWI), consistent with an infectious process such as hepatic microabscesses.

**Figure 3:**
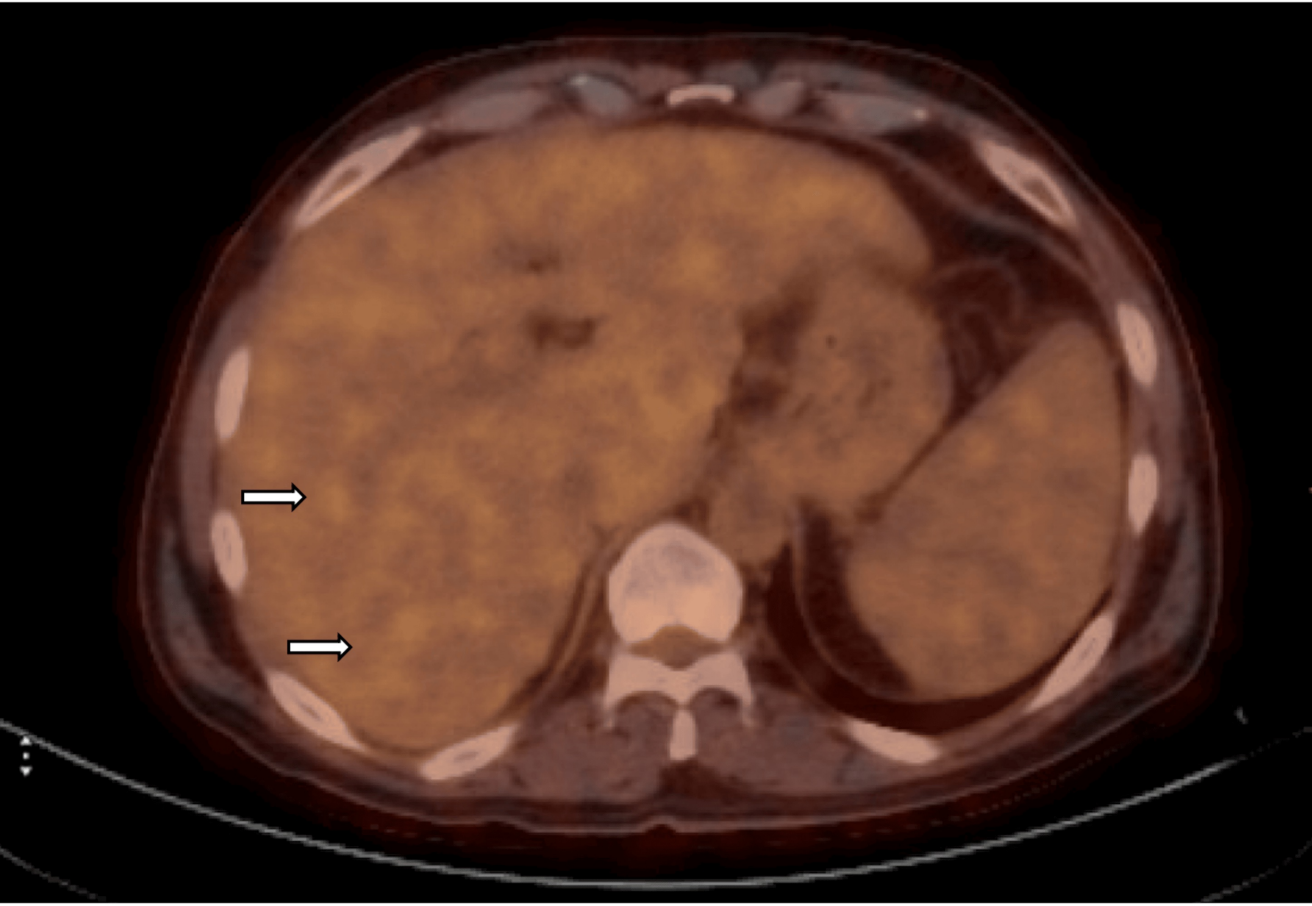
PET-CT axial images of the upper abdomen showing an absence of FDG uptake in the hepatic lesions (arrows), reducing suspicion for hepatic malignancy or lymphoma. FDG: fluorodeoxyglucose

A whole-body PET-CT (Figure 3) revealed no FDG (fluorodeoxyglucose) avidity within the hepatic lesions, arguing against metabolically active neoplasms or lymphoma.

An ultrasound-guided percutaneous liver biopsy was performed to further evaluate the hepatic lesions. Histopathological examination revealed advanced fibrosis (Laennec stage 3/4) and macrovesicular steatosis involving approximately 25% of hepatocytes. No ballooning cells, abscess formation, or malignancy were identified in the sampled tissue.

Further laboratory evaluation included serology for zoonotic infections. *Brucella melitensis* antibody was reactive with a high titer of 1:640, supporting the diagnosis of brucellosis in the context of compatible clinical and radiologic findings.

Following a multidisciplinary team discussion, a diagnosis of hepatic brucellosis was favored based on the clinical presentation, serological findings, and imaging characteristics. The patient was initiated on a combination antimicrobial therapy consisting of gentamicin for seven days, doxycycline, and trimethoprim-sulfamethoxazole (Bactrim). The latter two agents were continued for a prolonged course.

Follow-up and outcome

Following the initiation of targeted therapy for brucellosis, including gentamicin for seven days, doxycycline (ongoing), and trimethoprim-sulfamethoxazole (added later), the patient demonstrated notable clinical improvement. Fever subsided, and inflammatory markers and liver function tests began to trend downward (Table 2).

**Table 2:** Follow-Up Laboratory Evaluation Abbreviations: WBC, white blood cell count; Hgb, hemoglobin; PLT, platelet count; AST, aspartate aminotransferase; ALT, alanine aminotransferase; ALP, alkaline phosphatase; GGT, gamma-glutamyl transferase; CRP, C-reactive protein; ESR, erythrocyte sedimentation rate.

Laboratory Test	Follow-Up Value	Reference Range
White Blood Cell Count	4.4	4.0–11.0 × 10^9^/L
Hemoglobin	89.0	125–180 g/L
Platelet Count	168	150–450 × 10^9^/L
AST	33	5–40 U/L
ALT	34	0–41 U/L
ALP	123	40–129 U/L
GGT	213	8–61 U/L
CRP	2.52	0.0–6.0 mg/L
ESR	35	0–15 mm/hr
*Brucella melitensis* Antibody	Negative	<1:80 (Negative)

**Figure 4:**
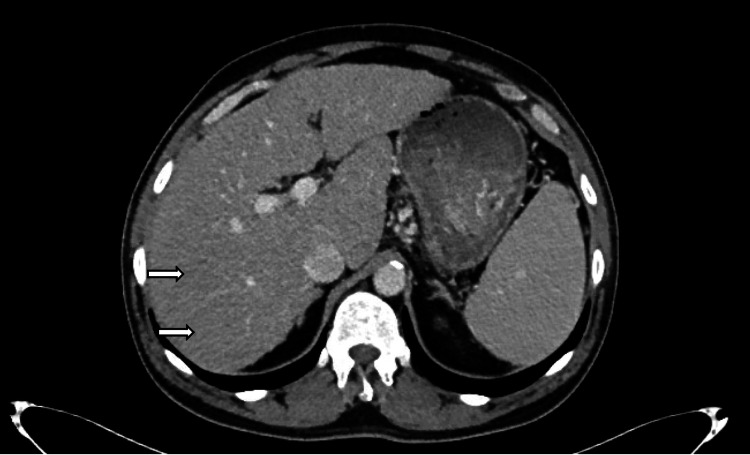
Follow-up contrast-enhanced CT of the abdomen and pelvis showing significant interval improvement in previously noted hepatic lesions. The liver demonstrates a cirrhotic morphology with no new or enlarging lesions. The ring-enhancing lesions have resolved, suggestive of a favorable response to antimicrobial therapy.

Eight weeks later, a follow-up contrast-enhanced CT of the abdomen and pelvis (Figure 4) revealed significant interval improvement of the previously identified ring-enhancing hepatic lesions, suggesting resolving infection or inflammation. No new or enlarging lesions were seen. The hepatic vasculature remained patent, with no biliary dilatation. Concurrently, repeat laboratory investigations showed improvement in inflammatory and hepatic markers (Table 2).

## Discussion

Hepatic involvement in brucellosis, although not uncommon, typically manifests as mild transaminase elevation or hepatosplenomegaly. However, focal hepatic complications, such as hepatic abscesses or brucellomas, are rare and diagnostically challenging, particularly in patients with underlying liver disease or atypical presentations. Our patient's case highlights the difficulty in diagnosing hepatic brucellosis in a cirrhotic liver, mimicking malignancy both radiologically and clinically.

Hepatic brucellomas represent chronic, localized granulomatous infections caused by *Brucella* species, typically appearing as space-occupying lesions that may mimic primary or metastatic liver tumors on imaging modalities such as CT or MRI [1] These lesions can be mistaken for hepatic malignancies or tuberculous granulomas due to their ring-enhancing appearance and non-specific features [2,3]. In our case, the multiplicity of small lesions and their radiological profile initially raised concern for infiltrative malignancy, especially in the setting of liver cirrhosis and elevated tumor markers.

Similar diagnostic confusion has been documented in the literature. For example, Sharif et al. described a case of brucellosis presenting with sepsis and cholestasis, initially suspected to be a hepatobiliary malignancy until serological and clinical correlation led to the correct diagnosis [4]. Similarly, Medvedeva et al. reported abnormal liver function as the first sign of brucellosis, with imaging features mimicking neoplastic or tuberculous lesions [5]. These cases, like ours, underscore the importance of considering brucellosis in the differential diagnosis of hepatic lesions, especially in endemic regions and in patients with relevant exposure history (e.g., raw milk ingestion, animal contact).

Histological confirmation of hepatic brucelloma is often elusive. In our patient, percutaneous liver biopsy failed to show abscess wall or granuloma formation, likely due to sampling error or the deep-seated nature of lesions, a limitation also reported by Sadia Pérez et al. and García Casallas et al., where biopsy results were either non-specific or negative for definitive features [6,7]. This adds to the diagnostic complexity, especially when cultures are also negative, as in our case.

Advanced imaging techniques offer valuable information but remain inconclusive in differentiating brucellomas from neoplastic lesions. While ring-enhancing lesions with diffusion restriction on MRI may raise suspicion for abscesses, they are not pathognomonic [8,9]. PET-CT, expected to distinguish infection from neoplasia, may also fail to show hypermetabolic activity in hepatic brucellomas, leading to false reassurance [10].

Multiple reports confirm that brucellar hepatic abscesses can mimic various pathologies. Starakis et al. described a case of liver abscess and pancytopenia initially thought to be hematologic malignancy [11], while Hamon et al. and Ennibi et al. documented hepatic lesions misinterpreted as metastatic or nodular neoplastic processes [12,13]. Koca et al. even reported a brucellar abscess presenting as an acute surgical abdomen [14].

Necrotizing forms of hepatic brucelloma with pseudo-tumoral features have been described in both radiologic and histopathologic correlations, further confounding diagnosis [15]. In chronic cases, lesions may remain indolent or masked, delaying treatment initiation. Amsilli et al. and Halimi et al. emphasized the importance of long-term follow-up and high clinical suspicion in such presentations [16,17].

Our case demonstrated clinical and radiological improvement after initiating anti-*Brucella* therapy with gentamicin, doxycycline, and later trimethoprim-sulfamethoxazole. Resolution of hepatic lesions on follow-up imaging without invasive intervention supports prior reports that medical therapy alone can be effective in selected patients [18,19].

## Conclusions

Hepatic brucellosis can mimic malignant or infectious hepatic lesions, particularly in cirrhotic patients. In endemic settings, clinicians should consider brucellosis when imaging is inconclusive and histology or cultures are nondiagnostic, integrating exposure history and serology to ensure timely diagnosis and treatment.
